# Role of fluid-phase complement system regulation in the development of hepatitis C virus-associated glomerulonephritis

**DOI:** 10.1371/journal.pone.0276017

**Published:** 2022-10-13

**Authors:** Sara T. Ibrahim, Marwa R. Abdelhamid, Neveen Lewis, Nahed Baddour, Ahmed G. Adam

**Affiliations:** 1 Department of Internal Medicine and Nephrology, Faculty of Medicine, Alexandria University, Alexandria, Egypt; 2 Renal Medicine, Kidney and Urology Centre, Alexandria, Egypt; 3 Department of Clinical Pathology, Faculty of Medicine, Alexandria University, Alexandria, Egypt; 4 Department of Pathology, Faculty of Medicine, Alexandria University, Alexandria, Egypt; National Institute of Infectious Diseases: Kokuritsu Kansensho Kenkyujo, JAPAN

## Abstract

**Objectives:**

It is not known why only some hepatitis C virus (HCV) infected patients develop glomerulonephritis (GN). Therefore, we investigated the role of soluble complement regulators in the development of HCV associated GN.

**Methods:**

Patients with HCV associated GN who were admitted to our nephrology unit between July 2016 and July 2018 were recruited to the study (group 1). Two other age and sex matched groups were studied as control groups: patients with HCV without GN (group 2) and healthy HCV negative volunteers (group 3). There were 26 participants in each of the three groups at the end of the recruitment period. An assay of serum fluid-phase complement regulators was performed using enzyme linked immunosorbent assay technique. Three complement single nucleotide polymorphisms (SNPs) were analyzed using real time polymerase chain reaction (Taqman; thermo fisher scientific): rs2230199 and rs1047286 for complement 3 (C3) and rs800292 for complement factor H (CFH).

**Results:**

Serum levels of complement 4 binding protein (C4BP) were significantly lower in group 1 (median 70 ng/ml) than in groups 2 (median 88.8 ng/ml) and 3 (median 82.8 ng/ml) with p value of 0.007. The minor allele (allele A) of rs800292 for CFH was significantly higher in group 2 and group 3 (G 54% and A 46%) than in group 1 (G 73% and A 27%), p = 0.04.

**Conclusions:**

Low C4BP levels are associated with GN in HCV infected patients. In addition, rs800292 SNP in CFH protects against GN in patients with HCV.

## Introduction

Hepatitis C virus (HCV) is a global health challenge, affecting about 58 million individuals worldwide with around 1.5 million new infections occurring per year [1]. Among all countries, Egypt has the highest infection rate in the world, with an estimated serological prevalence of 6.3% and a positive nucleic acid test of 4.4% [2].

In the general population, epidemiological studies have suggested that HCV seropositivity is linked to an increased risk for chronic kidney disease (CKD) and end stage renal disease (ESRD) [3, 4]. The association between HCV and CKD is potentially mediated by several mechanisms. First, HCV is associated with immune activation and immune complex glomerulonephritis (GN) [5]. Second, progressive liver disease, which is associated with longstanding HCV, may lead to hepatorenal syndrome. Lastly, HCV has been associated with an increased risk for diabetes [6], which is a leading cause of ESRD [7].

Hepatitis C virus infection has been reported in association with distinct histological patterns of GN in native kidneys. Membranoproliferative glomerulonephritis (MPGN) either associated with type II cryoglobulinemia, or without cryoglobulinemia, is the predominant type of HCV related GN [8]. Other less common GNs have also been reported in patients infected with HCV including membranous GN [9], focal segmental glomerular sclerosis [10], proliferative GN [9, 11], renal thrombotic microangiopathy associated with anticardiolipin antibodies [12], and fibrillary and immunotactoid glomerulopathies [13].

Hepatitis C virus associated MPGN is believed to be caused by the deposition of immune complexes in the glomeruli owing to persistent antigenemia. The immune complexes trigger the activation of the classical pathway of complement and the deposition of complement factors of the classical and terminal complement pathways in the mesangium as well as along the capillary walls [5]. There is also the potential for activation of complement by the alternative and lectin pathways in some patients in the setting of chronic infection and immune stimulation caused by HCV [14].

The activation of complement pathways is regulated by a variety of fluid phase (soluble) and membrane-associated proteins. For example, complement factor H (CFH), C4b-binding protein (C4BP), C1 inhibitor (C1INH), and complement factor I (CFI) are some of the soluble proteins known to regulate the activation of the complement system [15]. It is not known whether genetic polymorphisms involving factors H, B, membrane cofactor protein, and C3 that have been associated with complement mediated MPGN are also associated with HCV-associated MPGN [16, 17].

Based on the integral role of complement pathways in the development of immune complex mediated and complement mediated glomerular injury, we hypothesize that in addition to chronic HCV antigenemia, dysregulation in the complement pathways is also involved with and can predict the development of glomerular injury in patients infected with HCV. Therefore, we investigated the role of soluble complement regulators in the development of HCV-associated GN.

## Materials and methods

### Study population

**Fig 1:**
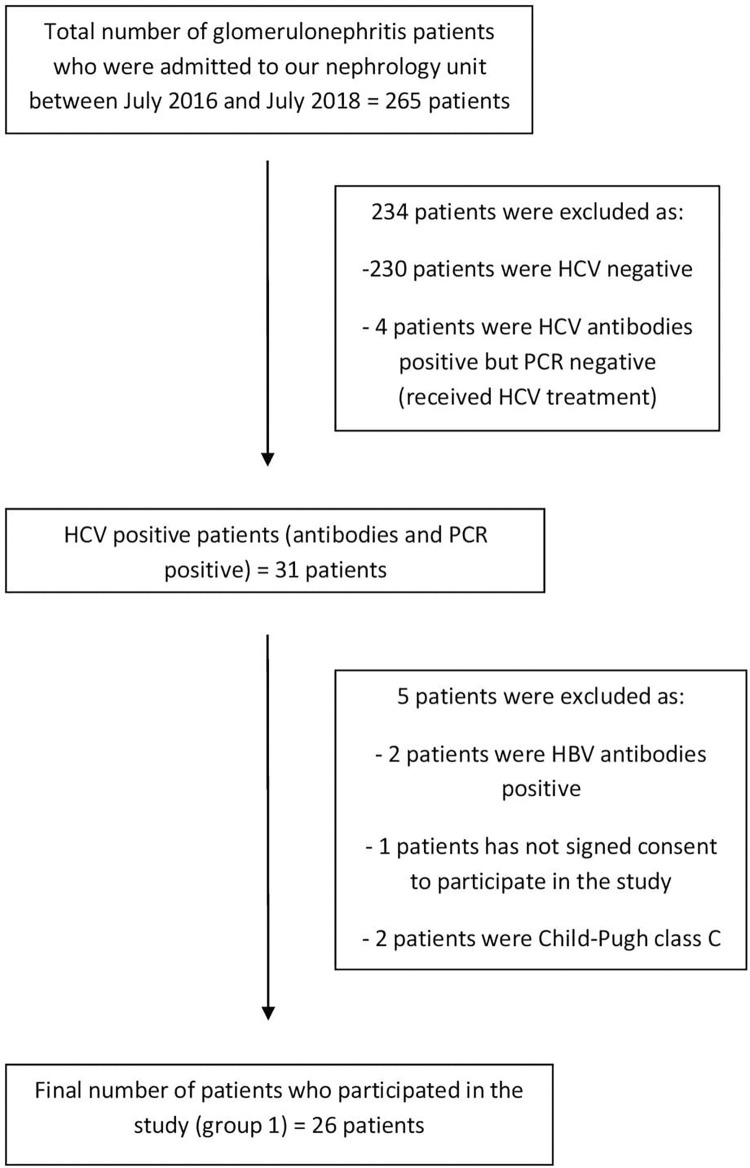
Flow chart for patients`recruitment to the study.

All patients with HCV associated GN who were admitted to the nephrology unit of our university hospital between July 2016 and July 2018 and who met the inclusion criteria but none of the exclusion criteria were recruited to the study (Fig 1). An equal number of age (with acceptance of the age range of +/- 5 years) and sex matched HCV positive patients without GN and healthy HCV negative volunteers were also recruited as control groups. Therefore, this study included three different groups that were defined as follows:

1. Group 1(HCV patients with GN): these patients were defined as having HCV (positive HCV antibodies and HCV quantitative polymerase chain reaction [PCR]) plus urinary protein creatinine ratio (uPCR) greater than 3 mg/mmol or estimated glomerular filtration rate (eGFR) less than 90 mL/min/1.73m^2^ with evidence of GN in renal biopsy or urine analysis suggested by the presence of active sediment inform of >5 RBCs and >5 WBCs per high-power field and/or cellular casts.
2. Group 2 (HCV patients without GN): these patients were defined as having HCV (positive HCV antibodies and HCV PCR) plus uPCR less than 3 mg/mmol and eGFR greater than 90 mL/min/1.73m^2^ with normal urine analysis defined as urine analysis with ≤ 5 RBCs and WBCs per high-power field and no casts or only hyaline casts.
3. Group 3 (healthy participants): these participants were defined as HCV negative (negative antibodies and HCV PCR) plus uPCR less than 3 mg/mmol and eGFR of 90 mL/min/1.73m^2^ or greater with normal urine analysis defined as urine analysis with ≤ 5 RBCs and WBCs per high-power field and no casts or only hyaline casts.

The study was conducted following the ethical guidelines of the 1975 Declaration of Helsinki and was approved by the ethical committee of The Faculty of Medicine, Alexandria University.

IRB approval number is 00008699.

Informed consent was obtained from all individual participants included in the study.

### Inclusion and exclusion criteria

All participants included in the study were older than18 years old and younger than70 years old.

Patients who had started antiviral therapy for HCV or immunosuppressive therapy for GN were excluded. In addition, patients with decompensated liver cirrhosis (Child Pugh class B or C) [18], who had co infection with hepatitis B virus (HBV) or human immunodeficiency virus (HIV) and who had chronic illness such as diabetes mellitus or hypertension with suspicion that their renal disease is due to their chronic illness were excluded.

### Clinical data and investigations

All participants were asked about their full medical history, including prior major illnesses, history of blood transfusion, serum creatinine and/or GFR history, and clinical diagnoses (e.g. diabetes).

At the time of enrollment to the study, the following investigations were conducted for all participants: blood urea, serum creatinine, eGFR calculated using the CKD-EPI 2009 formula (eGFR = 141xmin (SCr/κ,1)^α^ x max (SCr /κ,1)^-1.209^ x 0.993^Age^ x1.018 [if female]) [19], urine analysis, uPCR, serum albumin, international normalization ratio, total bilirubin, detection of serum HCV antibody, Hepatitis B surface antigen and HIV antibodies 1 and 2, HCV ribonucleic acid (RNA) quantitative PCR, rheumatoid factor (RF) and cryoglobulins. In addition, fundus examination to exclude diabetic or hypertensive retinopathy was conducted if needed.

### Measurement of serum level of complement factors

Blood was collected in tubes without anticoagulation then centrifuged at a speed of 2000 to 3000 rpm for 20 minutes. The supernatant (serum) was collected in multiple aliquots (to avoid repeated freeze-thaw cycles). The aliquots were stored at -80°C until the assay time. Assay of serum complement 3 and complement 4 levels was done by nephelometer (BN prospec system; Siemens Healthineers GmbH, Henkester 127, Germany). Assay of serum fluid-phase complement regulators (CFH, CFI, C4BP and C1 INH) was done by enzyme linked immunosorbent assay (ELISA) technique using SinoGeneClon Biotech CO., Ltd kits as follows: 10 microliters of the testing sample was added to 40 microliters of the diluted standard in every well. The wash buffer was diluted 30 fold with distilled water and was then used to wash all wells in the plate five times. Fifty microliters of horseradish peroxidase-conjugate reagent was then added to every well except the blank well. The plate was then incubated for 30 min at 37^⸰^ C and washed as previously. Fifty microliters of chromogen solution A and 50 microliters of chromogen solution B were then added to every well and incubated for 15 minutes at 37^⸰^ C, resulting in a blue color. Finally, 50 microliters of the stop solution was added to stop the reaction. After 15 minutes, absorbance was read at 450 nm and the results were calculated using the blank well as zero.

### Genotyping of single nucleotide polymorphisms in complement factors

Three single-nucleotide polymorphisms (SNP) were chosen: rs2230199 (R102G) and rs1047286 (Pro314Leu) for C3 and rs800292 (V62Ile) for CFH. These SNPs have been reported in the literature to be associated with or protective against complement mediated MPGN [16, 17].

Genomic deoxyribonucleic acid (DNA) was extracted using the PureLink® Genomic DNA Mini Kit (ThermoFisher Scientific, Rockford, IL, USA) in the immunogenetics laboratory of the Alexandria faculty of Medicine. Gene polymorphisms were detected by allelic discrimination using the 5`nuclease assay on a Rotorgene machine (Qiagen, Venlo, Netherlands) in the molecular biology laboratory. One ug genomic DNA was added to 10 ul of Taqman Genotyping Master mix 2X and 0.5 Um specific primers. Deionized water was added to bring the total volume to 20 ul. The thermal profile was: holding at 95^о^ C for 10 minutes, followed by 45 cycles of both denaturation; 95^о^ C for 15 seconds and Annealing/Extension: 60^о^ C for 1 minute. Interpretation of the results was performed where: VIC dye fluorescence or FAM dye fluorescence only denoted homozygous genotypes, while both fluorescence signals (VIC and FAM) denoted heterozygous genotype.

### Assessment of renal biopsies

Renal biopsies were offered to all HCV patients with GN at the time of admission and were performed only for those who accepted and signed informed consent. Specimens were examined by light microscope and were stained with the immune-peroxidase stains according to their availability in our pathology laboratory.

### Statistical analysis

Continuous data are expressed as medians and interquartile ranges (IQR). Categorical data are expressed as frequencies and percentages (%). For comparison between the three groups, the Kruskal-Wallis H test was used for the continuous variables and the chi-square test or Monte-Carlo test were used as appropriate for the categorical variables. For comparison between two groups, the Man-Whitney U test was used for the continuous variables and the chi-square test or Fisher`s exact test were used as appropriate for categorical variables. A p-value of less than 0.05 was considered statistically significant in all tests. All analysis was performed using SPSS version 23; (IBM Corp), licensed to Alexandria University.

## Results

### Characteristics of the studied groups

By the end of the two year recruitment period, 26 patients had been recruited to group 1. Twenty-six age and sex matched participants were also recruited to each of the other two groups.

The baseline clinical and biochemical characteristics of each group and a comparison between them are summarized in Table 1.

**Table 1:** Comparison between the studied groups according to the clinical and biochemical characteristics.

	Group 1 (HCV patients with GN) (n = 26)	Group 2 (HCV patients without GN) (n = 26)	Group 3 (Healthy participants) (n = 26)	P Value (Between the 3 Groups)	P Value (Between Group 1 & 2)	P Value (Between Group 1 &3)	P Value (Between Group 2 & 3)
**Age(Y),** median (IQR)	55 (48–60)	58 (48–65)	58 (50–64)	0.70	0.63	0.35	0.99
**Sex (female),** n (%)	16 (61.5%)	15 (57.7%)	15 (57.7%)	0.95	0.78	0.40	0.58
**Urea mmol/L,** median (IQR)	19 (13.5–27)	3.7 (2.8–5)	4 (2.7–5.5)	**<0.001**	**<0.001**	**<0.001**	0.08
**Creatinine μmol/L,** median (IQR)	230 (194–398)	53 (44–57.5)	44 (35–53)	**<0.001**	**<0.001**	**<0.001**	0.07
**eGFR**(CKD-EPI) ml/min/1.73m^2^	20 (15–24)	101 (94–115)	107 (104–122)	**<0.001**	**<0.001**	**<0.001**	0.07
**uPCR mg/mmol,** median (IQR)	30 (8–53)	0.5 (0.3–1)	0.2(0.1–0.5)	**<0.001**	**<0.001**	**<0.001**	**0.009**
**PT-INR,** median (IQR)	1.0 (1–1.1)	1.0 (1–1.2)	1.0 (0.8–1.0)	**0.002**	0.72	**0.002**	**0.002**
**Total bilirubin μmol/L,** median (IQR)	7 (5–12)	14 (10–15)	8.6 (5–12)	**<0.001**	**<0.001**	0.35	**0.001**
**Albumin g/L,** median (IQR)	25 (24–29)	33 (30–42)	38 (35–45)	**<0.001**	**<0.001**	**<0.001**	**0.03**
**HCV antibodies (positive)**, n (%)	26(100%)	26(100%)	0(0%)	**<0.001**	1.00	1.00	**<0.001**
**HCV RNA PCR (x10** ^**5**^ **) IU/mL**	3.0 (1.7–8.9)	5.0 (2.8–8.5)	-	^-^	0.37	-	-
**RF (positive)**, n (%)	15 (57.7%)	0 (0.0%)	0 (0.0%)	**<0.001**	**<0.001**	**<0.001**	**<0.001**
**Cryoglobulines (positive)**, n (%)	6 (23.1%)	1 (3.8%)	0(0.0%)	**0.02**	**0.04**	**<0.001**	**<0.001**

eGFR- estimated glomerular filtration rate calculated using the CKD-EPI equation, uPCR- urinary protein creatinine ratio, PT-INR- prothrombine time international normalization ratio, HCV RNA PCR- hepatitis C virus ribonucleic acid polymerase chain reaction, RF- rheumatoid factor, GN- glomerulonephritis, HCV- hepatitis C virus.

Continuous variables are expressed as median and interquartile range (IQR).The P-Value between the three groups is by Kruskal-Wallis H test and between two groups is by Man-Whitney U test.

Categorical variables are expressed as number (%). P value between the three groups is by chi-square test or Monte-Carlo test as appropriate and

between each two groups is by chi-square test or Fisher`s exact test as appropriate. P value is significant if less than 0.05.

The median age for group 1 was 55 years and females constituted 61.5% of the patients. There was no significant difference between the three groups regarding age and sex (p = 0.70). The median eGFR for group 1 was 20 ml/min/1.73m^2^ which was significantly lower than the eGFR of both groups 2 (median 101 ml/min/1.73m^2^) and 3 (median 107 ml/min/1.73m^2^) with p < 0.001. The uPCR of group 1 (median 30 mg/mmol) was significantly higher than both groups 2 (median 0.5 mg/mmol) and 3 (median 0.2 mg/mmol) with p < 0.001. There was no significant difference between groups 1and 2 regarding the quantitative PCR for HCV RNA with p value 0.37.

### Serum levels of complement factor

A comparison between the three groups regarding serum levels of the studied complements factors is summarized in Table 2.

**Table 2:** Comparison between the groups according to the serum levels of complement factors.

	Group 1 (HCV patients with GN) (n = 26)	Group 2 (HCV patients without GN) (n = 26)	Group 3 (Healthy participants) (n = 26)	P Value (Between the 3 Groups)	P Value (Between Group 1 & 2)	P Value (Between Group 1 & 3)	P Value (Between Group 2 &3)
**C3 mg/dl,** median (IQR)	75.5 (50–93)	108 (97–130)	142 (118–162)	**<0.001**	**<0.001**	**<0.001**	**0.001**
**C4 mg/dl,** median (IQR)	7 (2.1–15)	17 (13–26)	27 (23–36)	**<0.001**	**0.001**	**<0.001**	**0.001**
**CFH ng/ml,** median (IQR)	178.3 (157–230)	177 (144–237)	141.8 (135.5–171)	**0.03**	0.86	**0.02**	**0.02**
**CFI μg /ml,** median (IQR)	18.2 (16.5–26.5)	17.8 (13.5–21.5)	15 (12–18.5)	**0.02**	0.28	**0.004**	**0.04**
**C4BP ng/ml,** median (IQR)	70 (62–78.5)	88.8 (70.5–115)	82.8 (63–101)	**0.007**	**0.003**	**0.02**	0.46
**C1 INH pg/ml,** median (IQR)	884.8 (800–979)	862.5 (755.5–1044.5)	695.3 (629–970)	**0.01**	0.78	**0.007**	**0.01**

C3- complement 3, C4- complement 4, CFH- complement factor H, CFI- complement factor I, C4BP- complement 4 binding protein

C1 INH- complement 1 inhibitor, GN-glomerulonephritis, HCV- hepatitis C virus.

Variables are expressed as median and interquartile range (IQR). P value between the three groups is by Kruskal-Wallis H test and between two groups is by Man-Whitney U test.

P-Value is significant if less than 0.05

There were significant differences in serum levels of C3 and C4 between the three groups (p < 0.001). The lowest levels were found in group 1 (median for C3 = 75.5 mg/dl and for C4 = 7 mg/dl) and the highest levels were in group 3 (median for C3 = 142 mg/dl and for C4 = 27 mg/dl). Serum levels of CFH, CFI, and CI INH were significantly higher in group 1 (median 178.3 ng/ml, 18.3 μg/ml and 884.8 pg/ml respectively) and 2 (median 177 ng/ml, 17.8 μg/ml and 862.5 pg/ml respectively) than group 3 (median 141.8 ng/ml, 15 μg/ml and 695.3 pg/ml respectively) with p values 0.03, 0.02, and 0.01 respectively. However, serum levels of C4BP were significantly lower in group 1 (median 70 ng/ml) than groups 2 (median 88.8 ng/ml) and 3 (median 82.8 ng/ml) with p value 0.007.

### Genotyping of the SNPs in C3 and CFH

A comparison between the groups according to genotype variants and allele frequency of the studied SNPs is summarized in Table 3.

**Table 3:** Comparison between the groups according to genotype variants and allele frequency of the studied SNPs.

Genotypic variants	Group 1 (HCV patients with GN) (n = 26)	Group 2 (HCV patients without GN) (n = 26)	Group 3 (Healthy participants) (n = 26)	P value (Between the 3 Groups)	P value (Between Group 1 & 2)	P value (Between Group 1 & 3)	P value (Between Group 2 & 3)
**rs800292 (V62Ile) for CFH (G˃A)**							
GG	12(46.2%)	3(11.5%)	3(11.5%)				
AG	14(53.8%)	22(84.6%)	22(84.6%)	**0.002**	**0.02**	**0.02**	1
AA	0(0.0%)	1(3.8%)	1(3.8%)				
Allele frequency G	38 (73%)	28 (54%)	28 (54%)	0.06	**0.04**	**0.04**	1
A	14 (27%)	24 (46%)	24 (46%)				
**rs1047286 (Pro314Leu) for C3 (G˃A)**							
GG	0(0.0%)	2(7.7%)	3(11.5%)				
AG	24(92.3%)	23(88.5%)	22(84.6%)	0.62	0.61	0.41	0.90
AA	2(7.7%)	1(3.8%)	1(3.8%)				
Allele frequency G	24 (46%)	27 (52%)	28 (54%)	0.72	0.60	0.43	0.84
A	28 (54%)	25 (48%)	24 (46%)				
**rs2230199 (R102G) for C3 (G˃C)**							
GG	11(42.3%)	6(23.1%)	11(42.3%)				
CG	14(53.8%)	19(73.1%)	14(53.8%)	0.61	0.49	1	0.50
CC	1(3.8%)	1(3.8%)	1(3.8%)				
Allele frequency G	36 (69%)	31 (60%)	36 (69%)	0.50	0.31	1	0.31
C	16 (31%)	21 (40%)	16 (31%)				

CFH -complement factor H, C3- complement 3, GN- glomerulonephritis, HCV- hepatitis C virus.

P value is determined by chi-square test or Monte-Carlo test as appropriate

P value is significant if less than 0.05

There was no significant difference between the three groups regarding the genotype variants and allele frequency of rs1047286 (p value 0.62 and 0.72) and rs2230199 (p value 0.61 and 0.50) in C3. However, there was a significant difference regarding the genotype variants and allele frequency of rs800292 in CFH between group 1 (genotype variants; GG 46.2%, AG 53.8%, and AA 0% and allele frequency; G 73% and A 27%) and the other two groups (genotype variants; GG 11.5%, AG 84.6%, and AA 3.8% and allele frequency; G 54% and A 46%) with p value 0.02 and 0.04 respectively.

### Renal biopsy examination

Fifteen patients in group 1 had signed consent for the renal biopsy procedure. Using a light microscope, 11 of the 15 (73.3%) biopsies demonstrated features of MPGN. The remaining 4 biopsies (26.7%) showed mesangial proliferative changes without thickening of the glomerular basement membrane. With immunoperoxidase all biopsies showed IgM and IgG deposition in the mesangium and glomerular basement membrane of different degrees without any IgA deposition. Unfortunately, C3 and C4 staining were not available in our pathology laboratory.

## Discussion

Glomerular changes have been found by autopsy in approximately 55% of HCV-infected patients [20]. Until now, no study has succeeded to answer the question of why some of patients infected with HCV develop GN clinically, but others do not. In our study, we hypothesized that complement dysregulation may play a role in determining the higher susceptibility of some patients infected with HCV to develop GN. Thus, we measured serum levels of soluble complement regulators (CFH, CFI, C4BP, and C1 INH) and analyzed three SNPs in C3 and CFH to attempt to answer this question.

In our study, there was no significant difference in HCV quantitative PCR between groups 1 and 2. This suggests that the load of the virus (viremia) has no relation to the development of GN in chronic HCV patients. This was confirmed previously by Fabrizi et al. [21] who determined that even occult HCV can cause glomerular disease.

We found that serum levels of C3 and C4 were significantly lower in groups 1 and 2 compared with group 3, but the lowest levels were in group 1. This suggests that the cause of low C3 and C4 in GN patients is not only due to activation of the complement system and their involvement in the glomerular pathology but also there is a role for HCV infection itself in their low levels. This has been shown previously by Banerjee et al. [22] and Mazumdar et al. [23] in their research, as they showed a decrease in C3 mRNA and C4 mRNA levels in liver biopsies of patients with HCV which confirms the ability of HCV to down-regulate their transcription in the liver cells.

Hepatitis C virus modulates the complement system, trying to establish chronic infection and escape clearance by the immune system. Thus, it down regulates the expression of some complement factors such as C3, C4, C2 [24], and C9 [25]. On the other hand, for the same reason HCV up regulates the expression of CFH as was found by Kim et al. [24]. This agrees with our results, as serum levels of CFH were significantly higher in groups 1 and 2 than group 3. We also found the same for the other complement regulators, CFI and C1 INH, which seems logical and consistent with the trial of HCV to escape the clearance by the immune system. However, this did not agree with the finding of Kim et al’s study; which reported low levels of CFI mRNA in liver biopsies of patients infected with HCV compared with healthy subjects. This is a finding they could not explain and said it requires further exploration. To our knowledge, no study has investigated the relationship between HCV infection and C1 INH or C4BP serum levels or their transcription. We have found that C4BP serum levels in group 1 were significantly lower than its levels in both groups 2 and 3. This may suggest that the low C4BP serum levels are associated with GN in patients infected with HCV.

Abrera-Abeleda et al. [16] reported an association between C3 polymorphisms rs1047286 (Pro314Leu) and rs2230199 (R102G) and complement-mediated MPGN. This was explained by the weak ability of the polymorphic form of C3 to bind with CFH which increases the activity of the complement system, as reported by Heurich et al. [17]. In our study, we did not find an association between C3 polymorphism and GN in HCV-infected patients, but the small number of patients in our study may be the cause for absence of the association. However, we found that rs800292 (V62Ile), a SNP in CFH, is protective against the development of GN in HCV-infected patients (odd Ratio of having A allele, 0.43; confidence interval, 0.19–0.97; p = 0.0436). This SNP has been reported by Abrera-Abeleda et al. [16], Paun et al. [26] and Pickering et al. [27] to be protective against complement-mediated MPGN, age-related macular degeneration, and atypical haemolytic uremic syndrome respectively. Tortajada et al. [28] have found that the protective effect of the CFH allele A variant is owing to its better ability to bind C3b, compete with factor B in proconvertase formation, and enhance the inactivation of fluid-phase and surface-bound C3b. This confirms that there is no contradiction between our results as this polymorphism is a non-synonymous missense polymorphism that affects the function of CFH, not its serum level.

Our study was limited by the small number of the patients so we could not apply the Hardy-Weinberg equation. However, strengthened by the robust methodology, we recruited only patients who had not started antiviral or immunosuppressive therapy as literature has shown that these therapies may affect the complement system [29–31]. In addition, we excluded patients with mixed viral infection because the literature has suggested that other viruses affect the expression and level of complement factors [32]. Another limitation was the lack of renal biopsy in some HCV patients with GN, only 15 out of 26 patients agreed to do the renal biopsy, this raises questions about the cause of their renal disease. However, patients with chronic diseases such as diabetes mellitus and hypertension with the suspicion that their renal disease is due to their chronic illness were excluded.

## Conclusions

Low C4BP serum levels are associated with GN in patients infected with HCV. In addition, rs800292 (V62Ile) SNP in CFH protects against GN in patients with HCV.
